# Impact of Digitalization on Pediatric Practice and Childhood Health Care in Spain: Nationwide Survey Study

**DOI:** 10.2196/75310

**Published:** 2025-10-15

**Authors:** Alicia Santamaría-Orleans, Luis Ortiz-González, Maite Pérez-Hernández, Cristóbal Coronel-Rodríguez

**Affiliations:** 1 Scientific Communication Laboratorios Ordesa SL Barcelona Spain; 2 Biomedical Sciences Department Medicine and Health Sciences Faculty Universidad de Extremadura Badajoz Spain; 3 Medical Department Laboratorios Ordesa SL Barcelona Spain; 4 Pharmacology, Pediatrics and Radiology Department Faculty of Medicine Amante Laffón Health Care Centre Sevilla Spain

**Keywords:** health care digitalization, information and communication technology, ICT, pediatric ICT, artificial intelligence, AI, telemedicine

## Abstract

**Background:**

Health care digitalization and pediatric information and communication technology have facilitated the use of telemedicine and digital communication tools in pediatric practice, improving accessibility and efficiency. Meanwhile, artificial intelligence (AI) is emerging as a promising tool in medicine. However, the rapid adoption of these technologies has raised concerns regarding reliability and ethics.

**Objective:**

This study examines the level of digitalization in pediatric consultations and explores the perspectives of health care professionals (HCPs) on digital technologies in patient care, analyzing differences by age group, health care management, and institution type.

**Methods:**

An observational, cross-sectional survey was conducted among Spanish HCPs dedicated to pediatric care. Participants completed an 18-question web-based questionnaire evaluating their use of digital communication tools, perceptions of online health information, and opinions on AI in clinical practice. Statistical analyses compared responses across age groups, health care management type, and institution type.

**Results:**

A total of 495 pediatric specialists participated (female: 273/495, 58.2%; aged >45 y: 324/495, 69.8%). Most participants worked in urban settings (409/469, 87.2%), in primary care (243/313, 77.6%), and in the public sector (253/464, 54.5%). The telephone remained the most used communication channel (462/481, 96.1%), followed by email (290/481, 60.3%) and WhatsApp (139/481, 28.9%). Private-sector HCPs used digital platforms more frequently than public-sector HCPs, including email (136/206, 66% vs 135/247, 54.7%; *P*=.02), Instagram (23/206, 11.2% vs 5/247, 2%; *P*<.001), WhatsApp (105/206, 51% vs 26/247, 10.5%; *P*<.001), and Facebook (18/206, 8.7% vs 3/247, 1.2%; *P*<.001). Nearly all respondents (417/437, 95.4%) believed that parents were increasingly seeking health information online, yet a considerable proportion (158/458, 34.5%) reported that parents rarely consulted them about reliable sources. Overall, 85.4% (410/480) agreed that the internet and social media raise many questions among parents, while only 7.5% (35/465) believed that the information found is generally reliable. Nearly half of the participants (232/443, 48.5%) proactively suggested trustworthy digital resources, while 44.1% (211/443) did so only when asked. Younger respondents (*P*=.002), public-sector HCPs (*P*=.005), and primary care specialists (*P*=.004) were significantly more likely to offer this guidance, with scientific society resources being the most frequently recommended (402/430, 93.5%). We found that 78.6% (369/470) of participants were familiar with AI, with private-sector HCPs demonstrating greater knowledge than public-sector HCPs (*P*=.003). Overall, 59.6% (279/468) agreed that AI could significantly improve medicine, a view more commonly held by private-sector HCPs (*P*=.005). However, 94.8% (306/323) expressed ethical concerns, and 89.8% (422/470) wished to receive AI-related training.

**Conclusions:**

The survey highlights the increasing use of digital communication tools in pediatric practice, with private-sector HCPs leading adoption. While AI is viewed as promising, ethical dilemmas remain, underscoring the need for training. Limited confidence in online health information highlights the importance of strengthening digital literacy among both HCPs and parents to optimize patient care.

## Introduction

### Background

Technological development in recent decades has facilitated digital transformation, defined as the integration of digital solutions to create or modify existing organizational structures, resources, and social relationships [1,2]. Medicine has similarly undergone significant transformation through advances in this field. The adoption of innovative tools in health care, including pediatric information and communication technology (ICT), has expanded the provision of medical services via telecommunications, commonly referred to as telemedicine [3,4]. These services encompass telephone calls, instant messaging apps, videoconferencing, websites, mobile apps, and blogs [5]. These telemedicine options offer substantial benefits and have gained widespread user acceptance by facilitating access to information and enhancing physician-patient communication. In regions with limited health care accessibility, these solutions are particularly valuable, as they reduce travel constraints and optimize time utilization [6-8].

Although telemedicine had been steadily growing, it was the COVID-19 pandemic that triggered its rapid acceleration and widespread implementation [9]. Restrictions on hospital access during the pandemic created major challenges for patient monitoring and treatment adherence, prompting health care professionals (HCPs) to adopt digital solutions within online environments [10,11]. Unfortunately, the increasing use of the internet and social media by patients has generated new concerns. While these platforms can improve access to knowledge, not all content is reliable, which may result in the spread of misinformation. Thus, ensuring content quality control and appropriate regulation of these platforms is crucial [12,13]. In this context, HCPs could play a key role by leveraging social media as educational platforms when appropriate and guiding patients toward reliable sources of information [14].

More recently, the development of artificial intelligence (AI) has emerged as one of the most significant scientific and technological advances of our time, with applications extending across all sectors of society [15]. In medicine, AI is already being used to support specialists in various capacities, including big data analysis, medical image interpretation, workflow management systems, and clinical diagnostics. The implementation of AI has the potential to significantly improve the accuracy, efficiency, and overall quality of patient care [16-18]. Despite these benefits, AI lacks essential human attributes such as empathy and compassion, which are critical skills in health care. These limitations highlight the need for patients to feel that consultations remain under human oversight. As a result, ethical concerns regarding the use of AI continue to persist [19].

### Telemedicine in Pediatrics

Pediatrics is among the medical fields most influenced by new ICTs. Parents often seek immediate information and support, for which telemedicine and the internet offer convenient options [20]. During the COVID-19 pandemic, telemedicine use considerably increased in both ambulatory and hospital environments, suggesting that it is likely to become an integral component of routine pediatric care if such services remain available [21,22]. In Spain, most pediatricians reported using digital technologies to communicate with patients during the pandemic and believed that digital consultations would continue to play a role in the future [23].

Therefore, this survey aimed to examine and evaluate the digitalization of pediatric consultations and HCPs’ perceptions regarding the use of digital technologies in parenting.

## Methods

### Study Design

An observational, cross-sectional, descriptive survey was designed to obtain information on pediatric specialists’ perspectives regarding communication channels with patients, the quality of information available online, and the potential impact of AI in medicine. In addition, the survey analyzed and compared responses by age group, type of management, and type of institution to identify possible divergences within the system.

HCPs involved in pediatric care, from a sample of 5187 specialists registered in the database of Laboratorios Ordesa, a Spanish company specializing in nutrition and food supplements, were invited to participate. All individuals in this database had previously provided informed consent, in accordance with the Spanish Data Protection and Digital Rights Act, to be contacted by the company for informational purposes. An email invitation describing this study and containing a link to the questionnaire was sent to all members of this database, and the same invitation was also disseminated through the company’s social networks (X [formerly known as Twitter] and LinkedIn). No reminders were sent. Of the 5187 HCPs invited to participate, 495 (9.54%) completed the questionnaire. Explicit refusals were not recorded; thus, nonresponders were defined as those who did not reply to the invitation. Participants included HCPs from both the public and private sectors in Spain. No specific inclusion criteria were applied. Participants completed the questionnaire between September 2023 and June 2024.

The survey was developed by a team of HCPs with experience in questionnaire-based studies after a comprehensive literature review (Multimedia Appendix 1).

Since 2017, the research team has progressively explored key topics in communication between pediatricians and families through regular consultations with professionals in the field. This ongoing collaboration helped identify and refine the most relevant questions over time. The final structure and content of the questionnaire were based on a previously published survey that addressed similar topics [23]. In addition, a new section on AI was introduced, given the growing interest and limited existing data in pediatric settings. The questions in this section were developed with input from experts and inspired by recent findings in the field [24].

The questionnaire consisted of 18 questions across 3 sections. The first section included 7 items on sociodemographic data, including workplace location (rural vs urban). This classification was based on population size: rural (<2000 inhabitants), semiurban (2000-10,000 inhabitants), and urban (>10,000 inhabitants).

The second section focused on evaluating the digitalization of pediatric consultations and included 6 closed-ended questions measuring the level of agreement.

The third section assessed the impact of AI in clinical practice through 5 closed-ended questions. Responses regarding the influence of the internet and social media on parenting were rated on a scale ranging from 1 (completely disagree) to 10 (completely agree) and grouped into 4 categories according to Likert scaling: disagreement (1-3), neutral (4-6), agreement (7-10). In parallel, responses were analyzed by age group (29-45 y and >45 y), type of management (public or private), and type of institution (hospital or primary care).

### Data Analysis

Descriptive statistics (means and SDs) were calculated for quantitative variables, while frequencies and percentages represented categorical variables to describe the sample characteristics. The Fisher exact test was used to assess associations between qualitative variables, and the Mann-Whitney *U* test was used to compare quantitative variables between 2 independent groups. Statistical analyses were conducted in SAS (version 9.4; SAS Institute), with statistical significance defined as *P*<.05.

### Ethical Considerations

This study was exempt from formal ethics committee approval in accordance with the Spanish Organic Law 3/2018, of December 5, on the Protection of Personal Data and the Guarantee of Digital Rights. Participation was entirely voluntary. Pediatricians were informed about the objectives of the study, the voluntary nature of their participation, and the anonymous handling of their data prior to completing the questionnaire. Consent was implied through prior registration in the recruitment database and the voluntary completion of the online survey. No personally identifiable information was recorded, thereby ensuring participant privacy and confidentiality. The study did not involve patients, minors, or other vulnerable populations.

## Results

### Sociodemographic Data

Of the 5187 HCPs invited to participate, 495 (9.54%) completed the questionnaire. Of the 495 respondents, 273 (58.2%) were female, and the mean age was 52.2 (SD 10.9) years, with 324 (69.8%) aged more than 45 years. Most of the participants (409/469, 87.2%) worked in urban areas; 54.5% (253/464) were public-sector HCPs, while 77.6% (243/313) worked in primary care (Table 1).

**Table 1:** Sociodemographic characteristics of the study participants (N=495).

Characteristics			Values
**Sex (n=469), n (%)**			
	Male	196 (41.8)	
	Female	273 (58.2)	
Age (years; n=464), mean (SD)			52.2 (10.9)
**Age (y; n=464), n (%)**			
	29-45	140 (30.2)	
	>45	324 (69.8)	
**Type of management (n=464), n (%)**			
	Public	253 (54.5)	
	Private	211 (45.5)	
**Type of institution (n=313), n (%)**			
	Hospital	70 (22.4)	
	Primary care	243 (77.6)	
**Workplace location (n=469), n (%)**			
	Rural	11 (2.3)	
	Semiurban	49 (10.4)	
	Urban	409 (87.2)	
**Medical specialty (n=471), n (%)**			
	Pediatrics	407 (86.4)	
	Family medicine	31 (6.6)	
	Pediatric gastroenterology	15 (3.2)	
	Other	18 (3.8)	

### Digitalization of Pediatric Consultations and the Use of the Internet and Social Media

When asked about communication methods with patients, 96.1% (462/481) of the participants reported using the telephone, making it the most common channel. However, nearly 90% (429/481) also relied on ICTs such as email (290/481, 60.3%) and WhatsApp (139/481, 28.9%). Statistically significant differences were observed across subgroups: older HCPs (aged >45 y) were more likely to use WhatsApp (*P*<.001), while private-sector HCPs more frequently used digital communication methods, including email (*P*=.02), Instagram (*P*<.001), WhatsApp (*P*<.001), and Facebook (*P*<.001), compared to public-sector HCPs. By contrast, public-sector HCPs primarily relied on telephone communication (*P*=.01), and specialists working in hospitals used WhatsApp significantly more than those working in primary care (*P*=.002). These results are summarized in Table 2.

Regarding digitalization in information seeking, most of the respondents (n/N, 95.4%) believed that parents have increased their search for parenting information over the past year (Table 3). Table 4 presents the respondents’ recommendations on reliable digital sources of parenting information.

However, 34.5% (158/458) of the participants stated that parents had not consulted them about reliable digital sources of parenting information. The only significant difference was observed by age: younger specialists (aged 29-45 y) were more frequently asked about reliable sources of information (Table 5).

**Table 2:** Responses of health care professionals in pediatric practice (n=481) to the survey question “Which communication channels are you currently using to communicate with your patients?” Participants could select more than one option. *P* values were calculated with the Fisher exact test. Italicization indicates values that met the threshold for significance.

Communication channels	Total, n (%)	Age group (y), n (%)		*P* value	Type of management, n (%)			*P* value		Type of institution, n (%)			*P* value
		29-45 (n=136)	>45 (n=320)		Public (n=247)	Private (n=206)			Hospital (n=68)		Primary care (n=237)		
Telephone	462 (96)	129 (94.9)	309 (96.6)	.43	243 (98.4)	193 (93.7)	*.01* ^c^		62 (91)		229 (96.6)	.09	
Email	290 (60.3)	74 (54.4)	198 (61.9)	.15	135 (54.7)	136 (66)	*.02*		46 (68)		134 (56.5)	.12	
Blog	11 (2.3)	2 (1.5)	9 (2.8)	.52	3 (1.2)	8 (3.9)	.12		2 (3)		4 (1.7)	.62	
Twitter	6 (1.2)	1 (0.7)	5 (1.6)	.67	1 (0.4)	5 (2.4)	.10		1 (2)		3 (1.3)	.99	
Instagram	28 (5.8)	7 (5.1)	21 (6.6)	.67	5 (2)	23 (11.2)	*<.001*		7 (10)		14 (5.9)	.27	
WhatsApp	139 (28.9)	19 (14)	113 (35.3)	*<.001*	26 (10.5)	105 (51)	*<.001*		30 (44)		57 (24.1)	*.002*	
Facebook	21 (4.4)	6 (4.4)	15 (4.7)	.99	3 (1.2)	18 (8.7)	*<.001*		5 (7)		13 (5.5)	.56	
Other	62 (12.9)	21 (15.4)	39 (12.2)	.37	37 (15)	25 (12.1)	.41		8 (12)		33 (13.9)	.84	

**Table 3:** Responses of health care professionals in pediatric practice (n=437) to the survey question “Do you believe that in the past year, parents have increasingly sought information on child-rearing through digital media?” *P* values were calculated with the Fisher exact test.

Responses	Total, n (%)	Age group (y), n (%)		*P* value	Type of management, n (%)		*P* value	Type of institution, n (%)		*P* value
		29-45 (n=133)	>45 (n=297)		Public (n=235)	Private (n=198)		Hospital (n=63)	Primary care (n=231)	
Yes	417 (95.4)	129 (97)	282 (94.9)	.45	225 (95.7)	189 (95.5)	.99	63 (100)	225 (97.4)	.35
No	20 (4.6)	4 (3)	15 (5.1)	.45	10 (4.3)	9 (4.5)	.99	0 (0)	6 (2.6)	.35

**Table 4:** Responses of health care professionals in pediatric practice (n=430) to the survey question “If applicable, what types of sources do you recommend?” Participants could select more than one option. *P* values were calculated with the Fisher exact test. Italicization indicates values that met the threshold for significance.

Responses	Total, n (%)	Age group (y), n (%)		*P* value	Type of management, n (%)		*P* value	Type of institution, n (%)			*P* value
		29-45 (n=82)	>45 (n=148)		Public (n=143)	Private (n=85)		Hospital (n=26)	Primary care (n=131)		
I recommend my own sources	58 (13.5)	8 (9.8)	24 (16.2)	.23	13 (9.1)	20 (23.5)	*.004*	5 (19)	14 (10.7)	.32	
I recommend sources from the center where I work	66 (15.3)	13 (15.9)	34 (23)	.23	32 (22.4)	16 (18.8)	.62	3 (12)	30 (22.9)	.29	
I recommend sources from scientific societies	402 (93.5)	76 (92.7)	127 (85.8)	.14	126 (88.1)	74 (87.1)	.84	23 (89)	118 (90.1)	.73	
I recommend blogs and digital resources from other professionals	142 (33)	43 (52.4)	49 (33.1)	*.005*	52 (36.4)	39 (45.9)	.17	11 (42)	57 (43.5)	.99	
I do not recommend digital resources	3 (0.7)	1 (1.2)	6 (4.1)	.43	6 (4.2)	1 (1.2)	.26	0 (0)	0 (0)	—^a^	
Other	4 (0.9)	2 (2.4)	2 (1.4)	.62	3 (2.1)	1 (1.2)	.99	0 (0)	3 (2.3)	.99	

^a^Not applicable.

**Table 5:** Responses of health care professionals in pediatric practice to the survey question “Do parents ask you for reliable sources of information to learn about raising their children in the digital media?” *P* values were calculated with the Fisher exact test. Italicization indicates values that met the threshold for significance.

		Total (n=458), n (%)	Analysis by age (y), n (%)		*P* value		Analysis by type of management, n (%)				*P* value		Analysis by type of institution, n (%)					*P* value	
			29-45 (n=129)	>45 (n=305)			Public (n=236)		Private (n=198)				Hospital (n=65)		Primary care (n=227)				
**Responses**						*.03*						.42							.99
	Yes	300 (65.5)	95 (73.6)	191 (62.6)			150 (63.6)		134 (67.7)				45 (69)		158 (69.6)				
	No	158 (34.5)	34 (26.4)	114 (37.4)			86 (36.4)		64 (32.3)				20 (31)		69 (30)				

**Figure 1:**
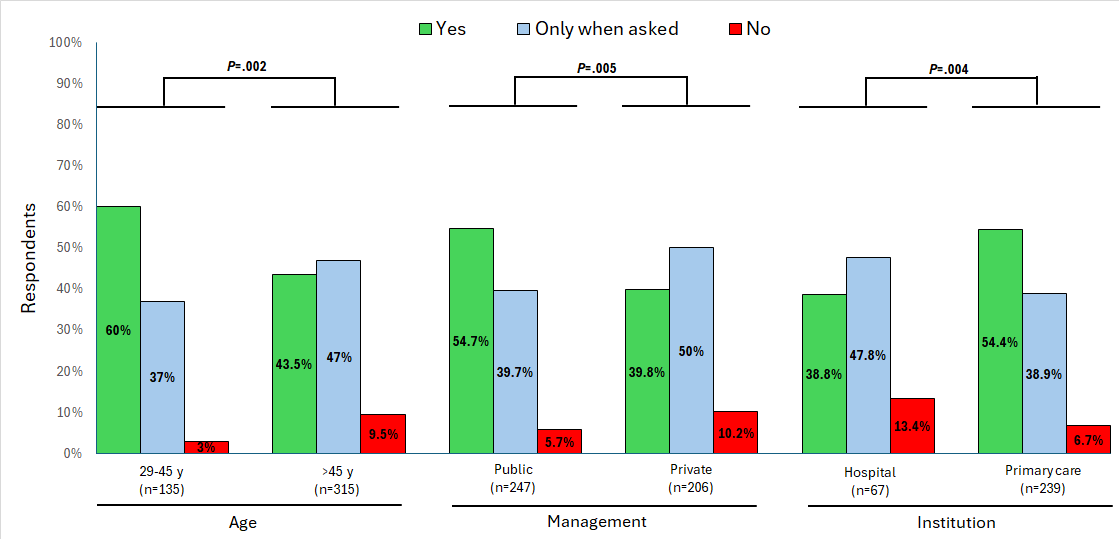
Proportions of health care professionals in pediatric practice who proactively recommend, recommend only when asked, or do not recommend trustworthy digital sources to parents, stratified by age group, type of management, and type of institution. Total responses: “yes”=48.5% (232/478); “only when asked”=44.1% (211/478); “no”=7.3% (35/478). The statistically significant *P* values (*P*<.05; Fisher exact test) are shown in bold.

When asked whether they recommend “reliable digital sources of parenting information,” 48.5% (232/443) of the HCPs responded that they normally do so even if parents do not ask, while 44.1% (211/443) provided recommendations only when asked. Grouped analysis indicated that younger respondents (*P*=.002), public-sector HCPs (*P*=.005), and primary care specialists (*P*=.004) were significantly more likely to provide such recommendations (Figure 1). The most frequently recommended sources were those from scientific societies, cited by 93.5% (402/430) of the HCPs, followed by blogs and digital content (142/430, 33%). Among the recommended options, younger professionals were more likely to propose blogs and digital content from other HCPs (*P*=.005), whereas private-sector physicians were more likely to recommend their own materials (*P*=.004; Table 4).

**Figure 2:**
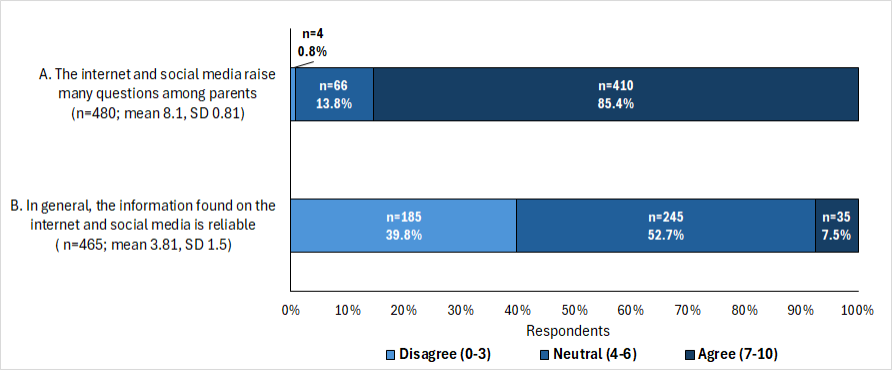
Perceptions of health care professionals in pediatric practice regarding the influence and reliability of the internet and social media in parenting. The bars represent the percentage of health care professionals who agreed, were neutral, or disagreed with each statement (A or B). The numbers indicate response counts. Mean, SD, and total responses are indicated for each statement.

For the statements “The internet and social media raise many questions among parents” and “In general, the information found on the internet and social media is reliable,” the mean scores were 8.10 (SD 0.81) and 3.8 (SD 2.0), respectively. The corresponding percentages are shown in Figure 2.

The only significant differences were observed by type of management, with private-sector HCPs being more likely to believe that the internet and social media raise many questions among parents (*P*=.03; Table 6). Table 7 presents the respondents’ perceptions on whether, in general, the information found on the internet and social media is reliable.

**Table 6:** Responses of health care professionals in pediatric practice to the survey question “The internet and social media raise many questions among parents.” Mann-Whitney U tests and Fisher exact tests were applied to calculate *P* values for quantitative and qualitative variables, respectively.

Variables			Analysis by age (y)					*P* value			Analysis by type of management					*P* value			Analysis by type of institution					*P* value
			29-45 (n=137)		>45 (n=315)					Public (n=248)			Private (n=207)					Hospital (n=68)			Primary care (n=240)			
Scores, mean (SD)			8.08 (1.80)		8.18 (1.74)		.68			8.01 (1.75)			8.31 (1.76)		.03			8.43 (1.51)			8.28 (1.68)		.70	
**Responses, n (%)**							.56								.09								.51	
	Disagree (1-3)	0 (0)		3 (1)					0 (0)			3 (1.5)					0 (0)			1 (0.4)				
	Neutral (4-6), n (%)	24 (17.5)		36 (11.4)					38 (15.3)			24 (11.6)					5 (7)			29 (12.1)				
	Agree (7-10), n (%)	113 (82.5)		276 (87.6)					210 (84.7)			180 (87)					63 (93)			210 (87.5)				

**Table 7:** Responses of health care professionals in pediatric practice to the survey question “In general, the information found on the internet and social media is reliable.” Mann-Whitney U tests and Fisher exact tests were applied to calculate *P* values for quantitative and qualitative variables, respectively.

		Age group			*P* value		Type of management, n (%)					*P* value			Type of institution, n (%)					*P* value
		29-45 (n=135)	>45 (n=302)			Public (n=235)		Private (n=205)					Hospital (n=69)			Primary care (n=241)				
Scores, mean (SD)		3.92 (1.85)	3.70 (2.04)	.16		3.61 (1.94)		3.99 (2.02)		.06			4.03 (1.94)			4.05 (1.84)		.67		
**Responses (y), n (%)**					.12						.77								.77	
	Disagree (1-3)	52 (38.5)	123 (40.7)			96 (40.9)		79 (38.5)					28 (41)			93 (38.6)				
	Neutral (4-6)	75 (55.6)	154 (51)			123 (52.3)		109 (53.2)					35 (51)			131 (54.4)				
	Agree (7-10)	8 (5.9)	25 (8.3)			16 (6.8)		17 (8.3)					6 (9)			17 (7.1)				

### Impact of AI in Medicine

**Figure 3:**
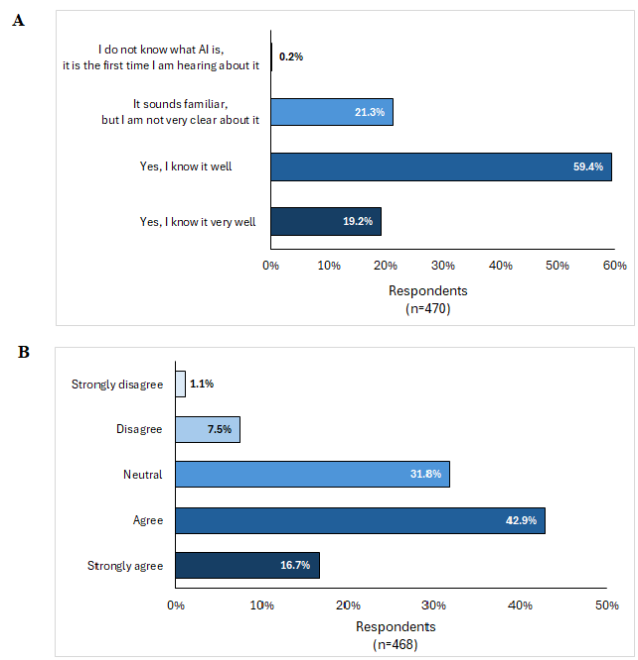
Health care professionals’ perspectives on artificial intelligence (AI) in pediatric practice. (A) Self-reported knowledge of AI among respondents (n=470), expressed as the percentage of participants in each category. (B) Level of agreement (n=468) with the statement “AI will significantly improve medicine.”

Participants were asked to share their opinions on recent advancements in AI and AI’s potential impact in medicine. Nearly 75% (369/470) of the HCPs reported knowing well (279/470, 59.4%) or very well (90/470, 19.2%) what AI is, while 21.3% (100/470) indicated that AI sounded familiar but that they were not very clear on what it entails (Figure 3A). Regarding the statement “I believe AI could lead to significant improvements in medicine,” more than half agreed (201/468, 42.9%) or strongly agreed (78/468, 16.7%), while 31.8% (149/468) remained neutral (Figure 3B). Regarding the medical areas where AI is perceived to offer the most improvements, respondents highlighted diagnostic assistance, medical training, and enhanced health care access in regions lacking specialists. The first 2 options were selected by approximately half of the HCPs (254/472, 53.8% and 228/472, 48.3%, respectively; Table 8).

Grouped analysis revealed several significant differences. Private-sector HCPs demonstrated greater knowledge of AI (*P*=.003; Table 9) and were more convinced than public-sector HCPs that AI could bring important advancements to medicine (*P*=.005), a view also strongly shared by hospital-based HCPs (*P*=.02; Table 10). Regarding areas of improvement, pediatric specialists aged more than 45 years were more likely to believe in AI’s potential to assist and contribute to diagnostic support (*P*=.02), as were hospital-based HCPs (*P*=.04); however, public-sector HCPs were more likely to believe that AI could enhance medical training (*P*=.04) and improve treatment adherence (*P*=.02; Table 8).

**Table 8:** Responses of health care professionals in pediatric practice (n=472) to the survey question “Select the medical areas where you think artificial intelligence (AI) could bring improvements.” Participants could select more than one option. *P* values were calculated with the Fisher exact test.

Responses	Total, n (%)	Age group (y), n (%)			*P* value		Type of management, n (%)			*P* value		Type of institution, n (%)			*P* value
		29-45 (n=136)	>45 (n=316)			Public (n=242)		Private (n=210)			Hospital (n=70)		Primary care (n=243)		
Medical training	228 (48.3)	63 (46.3)	156 (49.4)	.61		131 (54.1)		93 (44.3)	.04		29 (41)		91 (37.45)	.58	
Better access to health care in areas where specialists are unavailable	169 (35.8)	44 (32.3)	115 (36.4)	.45		96 (39.7)		70 (33.3)	.17		16 (23)		64 (26.3)	.64	
Diagnostic assistance	254 (53.8)	61 (44.9)	180 (57)	.02		127 (52.5)		120 (57.1)	.34		41 (59)		107 (44)	.04	
Better treatment adherence	78 (16.5)	18 (13.2)	55 (17.4)	.33		50 (20.7)		26 (12.4)	.02		9 (13)		28 (11.5)	.83	
Better patient monitoring	116 (24.6)	32 (23.5)	76 (24.1)	.99		62 (25.6)		49 (23.3)	.59		11 (16)		45 (18.5)	.72	

**Table 9:** Responses of health care professionals in pediatric practice to the survey question “You have most likely heard about ‘artificial intelligence (AI)’ recently, but do you know what AI actually is?” *P* values were calculated with the Fisher exact test. Italicization indicates values that met the threshold for significance.

			Age group (y), n (%)					*P* value			Type of management, n (%)						*P* value			Type of institution, n (%)						*P* value
			29-45 (n=133)		>45 (n=317)					Public (n=244)			Private (n=209)						Hospital (n=69)			Primary care (n=238)				
**Responses**								.70									*.003*									.44
	Yes, I know it very well	28 (21.1)		58 (18.3)					37 (15.2)			49 (23.4)						11 (16)			56 (23.5)					
	Yes, I know it well	75 (56.4)		195 (61.5)					142 (58.2)			128 (61.2)						45 (65.2)			130 (54.6)					
	It sounds familiar, but I am not very clear about it	30 (22.6)		63 (19.9)					65 (26.6)			31 (14.8)						13 (19)			51 (21.4)					
	I do not know what it is, it is the first time I am hearing about it	0 (0)		1 (0.3)					0 (0)			1 (0.5)						0 (0)			1 (0.4)					

**Table 10:** Responses of health care professionals in pediatric practice to the survey question “I believe that artificial intelligence (AI) could lead to significant improvements in medicine.” *P* values were calculated with the Fisher exact test. Italicization indicates values that met the threshold for significance.

			Age (y), n (%)				*P* value			Type of management, n (%)						*P* value			Type of institution, n (%)						*P* value	
			29-45 (n=135)		>45 (n=313)					Public (n=246)			Private (n=207)						Hospital (n=67)			Primary care (n=238)				
**Responses**								.64									*.005*									*.02*
	Strongly agree	27 (20)		50 (16)					32 (13)			44 (21.3)						26 (39)			49 (20.6)					
	Agree	55 (40.7)		137 (43.8)					96 (39)			99 (47.8)						30 (45)			131 (55)					
	Neutral	44 (32.6)		98 (31.3)					93 (37.8)			52 (25.1)						10 (15)			55 (23.1)					
	Disagree	7 (5.2)		25 (8)					22 (8.9)			10 (4.8)						1 (2)			3 (1.3)					
	Strongly disagree	2 (1.5)		3 (1)					3 (1.2)			2 (1)						0 (0)			0 (0)					

**Figure 4:**
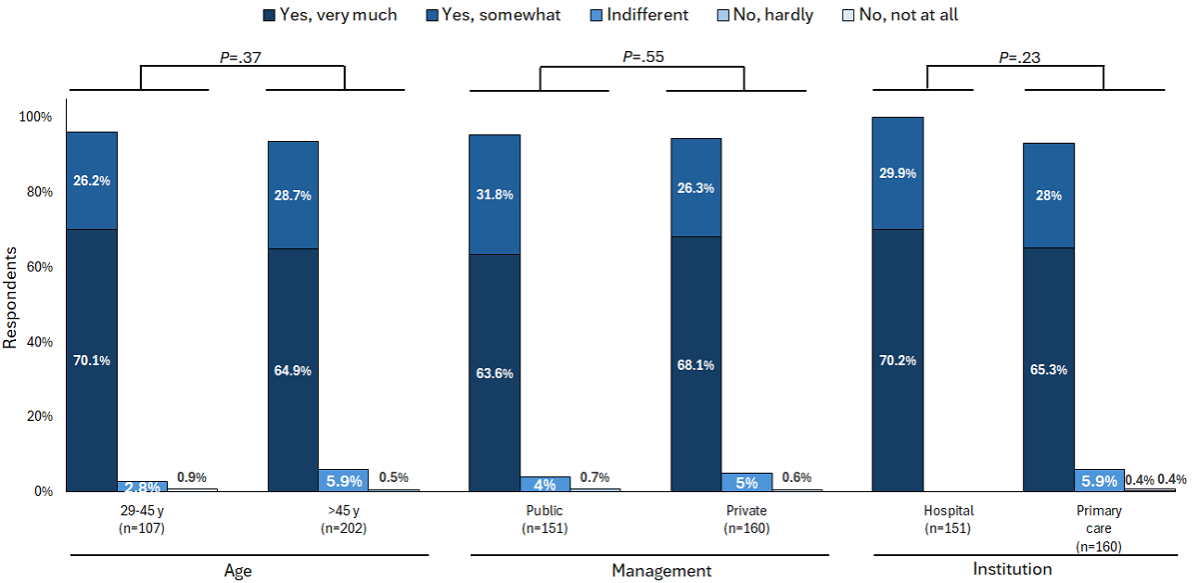
Health care professionals’ concerns regarding ethical and legal aspects of artificial intelligence. The bars represent the distribution of responses by age group, type of management, and type of institution. Total responses: “yes, very much”=66.3% (214/323); “yes, somewhat”=28.5% (92/323); “indifferent”=4.6% (15/323); “no, hardly”=0.3% (1/323); “no, not at all”=0.3% (1/323).

Regarding the ethical and legal aspects of AI, more than half of the participants admitted to being moderately (92/323, 28.5%) or highly (214/323, 66.3%) concerned, with no statistical differences between groups (Figure 4). In addition, most of the HCPs (422/470, 89.8%) stated that they would like to receive training on AI and its potential applications in medicine (Table 11), again with no significant group differences.

**Table 11:** Responses of health care professionals in pediatric practice (n=470) to the survey question “Would you like to receive training on AI and its potential applications in the field of medicine?” *P* values were calculated with the Fisher exact test.

		Total, n (%)	Age group (y), n (%)		*P* value		Type of management, n (%)				*P* value		Type of institution, n (%)				*P* value	
			29-45 (n=135)	>45 (n=316)			Public (n=247)		Private (n=206)				Hospital (n=67)		Primary care (n=240)			
**Responses**						.39						.34						.99
	Yes	422 (89.8)	119 (88.1)	288 (91.1)			219 (88.7)		189 (91.7)				62 (93)		220 (91.7)			
	No	48 (10.2)	16 (11.9)	28 (8.9)			28 (11.3)		17 (8.2)				5 (8)		20 (8.3)			

## Discussion

### Principal Findings

The integration of AI and digital tools is transforming medical practice, driving advancements in diagnostics, patient management, and health care accessibility [22]. In pediatrics, ICTs have become especially valuable, enhancing physician-parent communication [25] and providing immediate access to medical advice [26].

To the best of our knowledge, this survey is the first to examine the impact of health care digitalization across both public and private sectors and between hospitals and primary care services in Spain. It highlights a high prevalence of telephone use and digital communication tools such as WhatsApp and email among pediatric HCPs. These patterns may reflect the predominantly urban settings of the participants, as urban areas typically offer better digital infrastructure, high-speed internet, and more resources for digital health integration compared to rural regions [27]. Nevertheless, these results emphasize the widespread adoption of virtual communication models between parents and HCPs, consistent with previous reports from Europe [28,29].

Notable variations in digital tool use were observed, aligned with prior research indicating that adoption rates depend on the management system (public vs private) and the age of HCPs [30,31]. According to Rotaru and Edelhauser [30], private health care institutions generally implement digital solutions more quickly than public systems, which often encounter bureaucratic and financial barriers. Supporting this, the *Observatorio de Digitalización en la Sanidad Privada: Madurez Digital y Casos de Éxito* (*Digitalization Observatory in Private Health Care: Digital Maturity and Success Stories*) report underscores that private health care in Spain is leading the charge in digitalization, providing more accessible and personalized services [32]. Indeed, this is reflected in the higher use of digital platforms such as Instagram, WhatsApp, and Facebook by private-sector HCPs. It should be noted that in the Spanish public health care sector, the use of informal communication channels such as WhatsApp or Instagram between HCPs and patients is generally discouraged due to concerns regarding confidentiality, data protection, and professional boundaries [33].

Interestingly, private-sector HCPs preferred email communication more frequently than public-sector HCPs. This preference may be related to email’s ability to maintain formal records, align with structured consultation schedules, easily transfer digital images and documents, and facilitate asynchronous communication [34]. Nevertheless, telephone use remained dominant among public-sector HCPs, possibly due to centralized call systems or accessibility for families with limited digital resources. In addition, primary care physicians demonstrated a lower tendency to use digital tools overall compared to hospital-based HCPs, suggesting existing barriers or constraints in the use of digital health [35]. Notably, the Spanish Ministry of Health has implemented a digital health strategy aimed at transforming primary and community care in Spain, which is expected to help address these challenges [36].

Age has long been considered a key factor in the adoption of digital tools, with younger physicians typically seen as digitally literate and comfortable using digital platforms [31]. However, middle-aged adults (those aged 30-60 years) now also exhibit varying but significant levels of digital literacy, depending on their exposure to technological tools [31]. The simplicity and widespread use of WhatsApp, which has become an integral part of daily life for most people [37,38], likely explains its higher adoption among older HCPs.

Regarding parental use of digital media, most of the participants (417/437, 95.4%) reported that parents are increasingly turning to digital platforms for child-rearing advice. This finding aligns with previous research [39-41] indicating that social media is becoming a primary source of health information for both parents and HCPs [40]. This shift underscores the growing responsibility of HCPs to guide parents toward evidence-based resources. At the same time, concerns about misinformation have escalated, with many participants expressing skepticism about the reliability of online content, often due to the overwhelming volume of unverified information [25,40]. As a result, HCPs must strengthen their presence on social media, recommending curated and credible resources to counter misinformation and improve child health outcomes.

Most of the HCPs (402/430, 93.5%) reported recommending resources from scientific societies. This could be expected, as these institutions provide peer-reviewed, evidence-based information, which ensures content accuracy, reliability, and absence of commercial bias, making them trusted sources of medical advice [42]. Interestingly, private-sector HCPs, in addition to recommending scientific societies, were more likely than their public-sector counterparts to recommend their own online sources. This trend can be attributed to the fact that private hospitals often focus on increasing patient numbers and adherence [43]. Unlike their public-sector counterparts, who usually rely on institutional platforms and officially endorsed guidelines, private HCPs often operate in settings that require greater autonomy and visibility. Recommending personal or practice-specific resources may allow them to reinforce patient trust, provide tailored information, and enhance professional identity, while also compensating for the lack of centralized institutional tools available in the public system.

Evaluating how pediatric specialists are adapting to this digital era is critical for identifying areas of improvement and optimizing integration to ensure high-quality, patient-centered care. Indeed, the survey found that private-sector HCPs are generally more familiar with AI than their public-sector counterparts, likely due to greater investment in AI tools [32], more access to training [43,44], higher patient demand for technologically advanced services, and fewer bureaucratic obstacles to AI adoption [30].

Nearly half of the participants (82/135, 60.7%) believed that AI could significantly enhance medicine, especially in diagnostics and medical training. These results align with previous studies highlighting the role of machine learning in pediatric health care, particularly in supporting complex diagnoses and improving clinical decision-making [45]. However, this study identified differences in preferred areas of AI improvement: public-sector HCPs valued AI more for medical training and treatment adherence, whereas hospital-based HCPs (vs those working in primary care) prioritized diagnostic assistance. This trend was also seen among older HCPs. The reasons for these differences were not explored in the survey, but they may be explained by (1) AI’s role in continuing education, especially in underfunded public health systems; (2) AI’s potential to support treatment adherence—through automated reminders and multilingual resources—in populations considered vulnerable and attending public hospitals; (3) the ability of AI to automate monitoring; and (4) the potential to democratize access to therapeutic support tools in public health systems. Among older HCPs or those based in hospitals, the preference possibly reflects the need to address more complex or rare cases.

Previous studies have highlighted several concerns among HCPs regarding the integration of AI into health care. Key issues include fears of job displacement, particularly in specialties such as radiology, where AI may reduce the need for human workers. Such concerns are often linked to a lack of understanding of AI, with HCPs reporting superficial knowledge of this technology but still expressing skepticism about its potential. In addition, specialists lack trust in AI’s ability to address human emotions, which may degrade the patient-physician relationship, and they worry that overreliance on AI could undermine critical thinking, especially among younger professionals who may not have enough practical experience [46-49]. In this study, although approximately 90% (422/470) of the participants expressed interest in receiving AI training, concern about the legal and ethical aspects of AI was unanimous, regardless of age and type of management. Furthermore, a large proportion of respondents (30/133, 22.6%) reported neutral opinions regarding their knowledge of AI. These findings underscore the need for structured outreach, more reliable educational resources, and awareness campaigns on the role of AI in medicine.

Given the increasing role of digital tools and AI in pediatric care, integrating structured training programs for HCPs is essential to optimize their effective and ethical use. This could enhance health care efficiency, improve patient outcomes, and help address existing disparities among professional groups. Moreover, efforts should be directed toward establishing guidelines to ensure the reliability of online health information, given its growing influence on parental decision-making. Pediatricians should play a key role in this process by guiding families toward evidence-based resources.

Although this study focuses on Spain, its findings are in line with international trends in pediatric digitalization and AI adoption. Future research should explore cross-country comparisons to identify best practices and potential areas for policy improvement. In addition, further studies should evaluate the long-term impact of digitalization on pediatric health outcomes, as well as the effectiveness of AI-driven solutions in improving clinical decision-making and patient education.

### Strengths and Limitations

This study benefits from a large sample size that includes HCPs from different regions, age groups, health care settings, and professional backgrounds, providing a broad overview of current practices and perceptions regarding digital communication and AI in pediatrics. The questionnaire was specifically designed to capture both the use of digital tools and attitudes toward AI, and the stratified analyses offer valuable insights into differences by age, type of management, and type of institution. Although digital health technologies evolve rapidly, the survey was conducted over a relatively short period (September 2023-June 2024), making it unlikely that substantial shifts in professional perceptions occurred during data collection.

However, some limitations should be acknowledged. Recruitment through Laboratorios Ordesa’s database and social media channels may have introduced selection bias, favoring HCPs with higher digital literacy or stronger professional ties to the private sector. In addition, the relatively low response rate (495/5187, 9.54%) raises the possibility of nonresponse bias, as participants who completed the survey may differ from nonrespondents in their engagement with digital tools or interest in AI. This could limit the generalizability of the findings to those less engaged with digital platforms. To mitigate this, the study sought participation from professionals across diverse health care settings and sectors, aiming to capture a heterogeneous sample.

Reliance on self-reported data may have introduced recall bias or social desirability bias, potentially leading to overestimation of AI knowledge or frequency of digital tool use. The cross-sectional design also limits the ability to establish causal relationships between variables, as it captures data at a single point in time. Nonetheless, the survey provides a valuable snapshot of current perceptions and practices among HCPs, allowing for the identification of relevant trends and areas for future longitudinal research. In addition, rural HCPs were underrepresented (11/469, 2.3%), which may limit the applicability of the results to rural practice contexts, where resources, infrastructure, and digital adoption present greater challenges. Public-sector participants were also underrepresented, which may not fully reflect their predominance in Spain [50]. There are no similar published works with which our data can be directly compared. Finally, the absence of a mandatory requirement to respond to all questions may have introduced some data gaps, potentially biasing the results.

### Conclusions

The findings of this study highlight a significant level of digitalization in pediatric consultations, with widespread adoption of ICTs among pediatric specialists, particularly in urban settings. In addition, AI knowledge among HCPs seems to be relatively high, with many recognizing its potential benefits in diagnostics and medical training. However, concerns about misinformation as well as the ethical and legal aspects of AI continue to persist.

To enhance health care efficiency, improve patient outcomes and education, and help address existing disparities among professional groups, structured training programs on digital tools and AI, together with guidelines to ensure the reliability of online health information, are warranted.

## Appendix

Multimedia Appendix 1 Clinical practice questionnaire.

## Abbreviations

AI: artificial intelligence
HCP: health care professional
ICT: information and communication technology

## Data Availability

The datasets generated and analyzed in this study are available from the corresponding author on reasonable request.
