# Supplementary material for: Impact of Digitalization on Pediatric Practice and Childhood Health Care in Spain: Nationwide Survey Study
Source: JMIR Pediatr Parent. 2025 Oct 15;8:e75310. doi: 10.2196/75310 (PMC12572743; doi:10.2196/75310)
Supplement: Multimedia Appendix 1 [file pediatrics_v8i1e75310_app1.docx]

**Table S1.** Clinical Practice Questionnaire

| **Sociodemographic data** | |
| --- | --- |
| 1 | Sex: -Male - Female |
| 2 | Age:_____ |
| 3 | Region of professional practice:_____ |
| 4 | Type of facility:  -Hospital  -Primary care |
| 5 | Type of management:  -Public  -Private |
| 6 | Location of the workplace:  -Rural (<2000 pop.)  -Semi-urban (2000<n<10,000 pop.)  -Urban (>10,000 pop.) |
| 7 | Medical specialty:  -Pediatrics  -Pediatric gastroenterology  -Family medicine  -Other:_____ |
| **Digitalization of pediatric consultations** | |
| 1 | Which communication channels are you currently using to communicate with your patients? *Select all that apply*  -Telephone -Mail -Blog -Twitter -Instagram  -WhatsApp -Facebook -Other:_____ |
| 2 | Do you believe that in the past year, parents have increasingly sought information on raising their children through digital media?  -Yes -No -NS/NC |
| 3 | Do parents ask you for reliable sources of information to learn about raising their children in the digital media?  -Yes -No -NS/NC |
| 4 | Do you recommend trustworthy sources where parents can find information on raising their children in the digital media?  -Yes -No -Only when asked -NS/NC |
| 5 | If applicable, what types of sources do you recommend? *Select all that apply*  -I recommend my own sources  -I recommend sources from the center where I work  -I recommend sources from scientific societies  -I recommend blogs and digital resources from other professionals  -Other:_____ |
| 6 | Do you agree with the following statements? *Rate on a scale from 1 to 10, where 1 means completely disagree and 10 means completely agree.*   - The internet and social media raise many questions among parents - In general, the information found on the internet and social media is rigorous |
| **Impact of AI in clinical practice** | |
| 1 | You have most likely heard about "Artificial Intelligence (AI)" recently, but do you know what AI actually is?  -Yes, I know it very well  -Yes, I know it well  -NS/NC  -It sounds familiar, but I am not very clear about i  -I do not know what AI is, it is the first time I am hearing about it |
| 2 | I believe that AI could lead to significant improvements in medicine.  -Strongly agree -Agree -Neutral -Disagree -Strongly disagree |
| 3 | Select the medical areas where you think AI could bring improvements. *Select all that apply*  -Medical training  -Diagnostic assistance  -Better access to health care in areas where specialists are unavailable  -Better adherence to treatment  -Better patient monitoring |
| 4 | I am concerned about the ethical and legal aspects related to the use of AI  -Yes, very much  -Yes, somewhat  -Indifferent  -No, hardly  -No, not at all |
| 5 | Would you like to receive training on AI and its potential applications in the field of Medicine?  -Yes -No |
